# Accuracy of CEUS-guided sentinel lymph node biopsy in early-stage breast cancer: a study review and meta-analysis

**DOI:** 10.1186/s12957-020-01890-z

**Published:** 2020-05-29

**Authors:** Qiuxia Cui, Li Dai, Jialu Li, Jialei Xue

**Affiliations:** Department of Thyroid and Breast Surgery, Changshu Hospital Affiliated to Nanjing University of Chinese Medicine, Suzhou, China

**Keywords:** Breast cancer, Contrast-enhanced ultrasound, Microbubble, Sentinel lymph node, Sentinel lymph node biopsy, Diagnostic accuracy, Review, Meta-analysis

## Abstract

**Objective:**

To investigate whether preoperative localization of sentinel lymph node (SLN) by contrast-enhanced ultrasound (CEUS) can further improve the accuracy of sentinel lymph node biopsy (SLNB).

**Method:**

Collect published literatures or conference reports by searching electronic databases. The Quality Assessment of Diagnostic Accuracy Studies-2 (QUADAS-2) evaluation method is used to evaluate the quality of the screened literatures. The pooled risk ratio of cancer metastasis of SLN identified by CEUS (CE-SLN) compared with SLN not identified by CEUS (nonCE-SLN) is calculated, and the pooled diagnostic accuracy of CE-SLN for pathological status of all SLNs is also evaluated.

**Result:**

Through search and screening, a total of 16 studies were included, of which five and seven studies, respectively, entered the meta-analysis of metastatic risk ratio and diagnostic accuracy. The localization rate of preoperative CEUS for sentinel lymph nodes was 70 to 100%. The meta-analysis shows that the risk of metastasis of SLN identified by CEUS is significantly higher than that not identified by CEUS, 26.0% vs 4.6%, and risk ratio (RR) is 6.08 (95% CI 4.17–8.85). In early-stage breast cancer, the pathological status of CE-SLN is a good representative of all SLNs, with a pooled sensitivity of 98% (95% CI 0.94–1.00), pooled specificity of 100% (95% CI 0.99–1.00), diagnostic odds ratio (DOR) of 2153.18 (95% CI 476.53–9729.06), and area under the subject receiver operating characteristic (SROC) curve of 0.9968.

**Conclusion:**

In early-stage breast cancer, preoperative localization of SLN by CEUS is expected to further improve the accuracy of sentinel lymph node biopsy (SLNB).

## Introduction

The latest statistics of world cancer showed that breast cancer is still the world’s highest incidence and mortality of female malignant tumors [1]. With the improvement of cancer prevention awareness in the population and medical diagnosis technology, the majority of breast cancers have been found at an early stage, clinical axillary lymph node (ALN) negative [2]. For these patients, the clinical benefits of axillary lymph node dissection (ALND) are very limited, and the serious complications of upper extremity lymphedema caused by axillary lymph node dissection cannot be ignored [3, 4, 8]. Therefore, the guidelines consider sentinel lymph node biopsy (SLNB), which combines the accuracy of axillary staging with less surgical trauma, as the standard axillary management for clinical axillary negative patients [4–8]. Sentinel lymph node (SLN) is defined as the first station of lymph node which arrives from the breast lesion to the axillary lymphatic drainage, and the pathological status of this lymph node may indicate the pathological status of the total axillary lymph nodes [9, 10]. Currently, common clinical lymphatic mapping methods for sentinel lymph node biopsy include isotope and/or blue dye. A meta-analysis showed that the detection rate of sentinel lymph nodes by blue dye alone was 83%, while that by isotope alone was 89% [9]. The combination of isotope and blue dye can increase the success rate to 96 ~ 99% [4, 11]. However, due to higher medical costs and inevitable radiation exposure and contamination problems, the clinical application of isotope is limited, especially in developing countries. The blue dye also has some adverse reactions, including local skin reactions, tattoo effects, and more serious allergic reactions. In addition, the dual-tracer method also has some disadvantages, such as time-consuming and complicated operation steps. Therefore, some scholars began to explore a new sentinel node tracer technology [12, 13].

### Ultrasonography and contrast-enhanced ultrasound

Conventional axillary ultrasonography is the most commonly used auxiliary examination for preoperative evaluation of lymph node status, with a high detection rate for suspicious axillary lymph nodes, but for patients with no or only micrometastasis of axillary lymph nodes, the accuracy of lymph node location and imaging is relatively low [14, 15]. Moreover, conventional gray-scale ultrasound could not determine whether the lymph node was sentinel node. Because of the high operability of ultrasound and the high identification rate of suspicious lymph nodes, many researchers choose to explore new sentinel lymph node tracing method to assist ultrasonic technology. In 2004, Goldberg et al. [16] successfully used contrast-enhanced ultrasound technique to trace lymphatic drainage pathway and sentinel lymph node in a swine model with melanoma, and the detection rate reached 90%. Omoto et al. took the lead in the clinical study of sentinel node tracer with contrast-enhanced ultrasound in breast cancer patients [17]. The first ultrasound contrast agent used was 25% albumin, with a relatively low success rate. With the emergence of a new generation of microbubble contrast agents, such as SonoVue and Sonazoid, the operability and diagnostic timeliness of ultrasonic microbubble imaging technology have been greatly improved due to their good stability and sufficient dispersion time [18–20]. Sever et al. [21] and Omoto et al. [22] used SonoVue and Sonazoid, respectively, to successfully trace and locate sentinel lymph nodes under the guidance of ultrasonography and contrast-enhanced ultrasound (CEUS).

In the past decade, a number of clinical studies had explored the identification of sentinel lymph node in early-stage breast cancer by contrast-enhanced ultrasound and its application value in sentinel lymph node biopsy. Some studies showed that CEUS can further improve identification of SLN and reduce SLNB surgical trauma [23–26]. However, some studies suggested that the false negative rate of SLNB guided by CEUS alone is high and the diagnostic accuracy is low [27, 28]. Since the difference in accuracy of assessing axillary status between CEUS-guided SLNB and conventional SLNB is not clear, this meta-analysis reviews the reported studies focusing on SLNB guided by CEUS, further analyzes the difference in the positive rate between SLNs identified by CEUS and SLNs identified not by CEUS, and also analyzes the accuracy of SLN identified by CEUS in diagnosing the pathological status of overall axillary sentinel nodes, in order to explore whether CEUS can further improve the accuracy of sentinel lymph node biopsy.

## Materials and methods

### Literature search

We searched the following databases, MEDLINE, EMBASE, Cochrane library, PubMed, and Web of Science, by using keywords. The retrieval time is limited to before November 31, 2019. Keywords and search methods are as follows: (“breast cancer” or “breast tumor” or “breast neoplasm”) and (“sentinel lymph node” or “SLN” or “axillar sentinel lymph node” or “sentinel lymph node biopsy” or “SLNB”) and (“CEUS” or “contrast-enhanced ultrasound” or “microbubble” or “contrast agent”), and by reading the references of the identified literature, potentially relevant literature can be found.

### Inclusion and exclusion criteria

The inclusion criteria are as follows: (1) preoperative use of CEUS to identify SLN; (2) SLN positioning mark (without limitation of positioning method); (3) intraoperative SLNB was performed by dye method (including fluorescence) and/or nuclide method; (4) histopathological analysis of lymph nodes was used as the gold standard; (5) the study subjects were breast cancer patients over the age of 18, regardless of gender; and (6) prospective and retrospective studies.

The exclusion criteria are as follows: animal studies, non-breast cancer studies, intravenous microbubble contrast agent (not for SLN), review, and subjects repeat.

### Data extraction and analysis

**Fig. 1:**
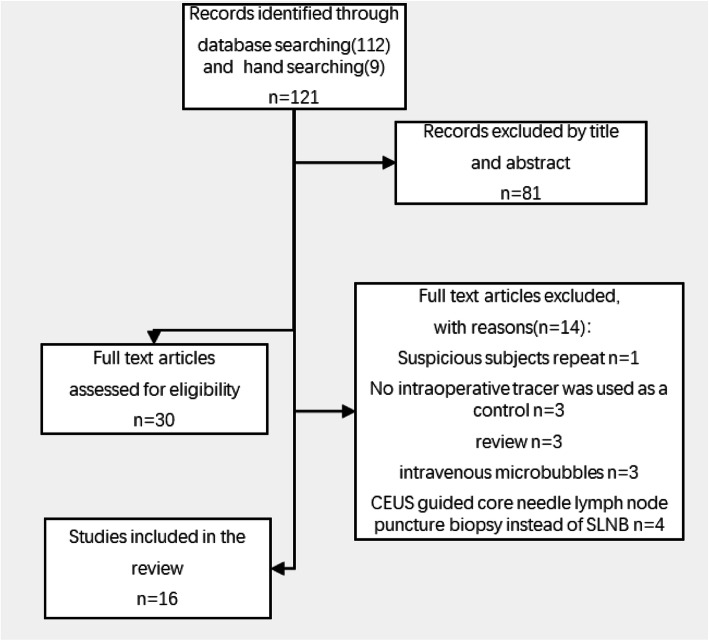
The selection process of the relevant studies

The contents of the literatures were evaluated by two authors of this manuscript. The author, LD, evaluated the relevance of the studies on the basis of the inclusion criteria and excluded the articles whose titles and abstracts clearly indicated that they were not relevant to the review [29, 30]. The full text of the research relevant to the topic was obtained and further screened by the author LD. These literatures were independently verified by two authors, LD and JL. When the authors have different opinions, consensus was reached through discussion. The selection process of the relevant studies is shown by the PRISMA flow chart (Fig. 1) [31].

The extracted data include the study design; countries; recruitment procedures; settings; inclusion and exclusion criteria; number of patients; research purpose; demographic characteristics, such as age, sex, tumor size, invasive, or ductal carcinoma in situ (DCIS); information on interventions, such as contrast agents used, imaging systems, and injection methods; description of comparator (isotopes and/or blue dyes); and details regarding the outcome measurement and results. Details include the following: the identification rate of SLN by CEUS, the total number and metastatic number of SLN identified by CEUS (CE-SLN) and SLN not identified by CEUS (nonCE-SLN), the median number of SLN identified by different methods in individual patient, and the pathological result of CE-SLN and nonCE-SLN in individual patient who successfully identified SLN by CEUS that contains true positive—CE-SLN pathological positive; true negative—CE-SLN negative and nonCE-SLN negative; and False negative—CE-SLN negative but nonCE-SLN positive.

Detailed data were extracted from the included studies and entered into Review Managers software 5.3(http://www.tech.cochrane.org/revman/download) [32]. The metastasis risk ratios for two sets of different SLNs and 95% confidence interval were calculated by the fixed or random-effects models based on heterogeneity between studies, and showed in a forest diagram [32]. The clinical and methodological heterogeneity of the studies was evaluated by observing the characteristics of the studies and visually evaluating the forest plots [33]. Statistical heterogeneity among studies was assessed by chi-square test, and the effect of true variability was emphasized by calculating *I*-square statistics, and the *I*^2^ statistic values of < 30%, 30–70%, and ≥ 70% were considered as mild, moderate, and severe heterogeneity, respectively [34]. Meta-disc 1.4 (http://www.hrc.es/investigacion/metadisc_en.htm) was used for meta-analysis of diagnostic accuracy [35]. The pooled accuracy (sensitivity, specificity) and 95% confidence intervals of CE-SLN in diagnosing the pathological status of overall axilla sentinel nodes were evaluated. And in this review, all studies used the same pathological diagnostic threshold, containing micrometastasis (defined as lymph node metastases with a diameter of less than 2 mm but greater than 0.2 mm) and macrometastasis (defined as lymph node metastases with a diameter of more than 2 mm), so threshold effects were not an issue [36].

### Quality control and bias

In each of the articles in this review, the Quality Assessment of Diagnostic Accuracy Studies-2 (QUADAS-2) evaluation method was used to assess the risk of bias [37, 38]. Two reviewers assessed the quality of the included studies, and any differences were resolved by consensus.

## Result

A total of 121 reports were found in the search. After preliminary reading of the titles and abstracts, 81 irrelevant articles were excluded, and the remaining 30 reports were screened for the next step. Sixteen studies were finally selected, and the detailed steps of literature screening and the detailed exclusion of 14 literatures are illustrated in the PRISMA diagram (Fig. 1). All 16 studies used CEUS in combination with conventional methods (dye and/or nuclide) to perform sentinel lymph node biopsy. Seven studies which detailed the diagnostic accuracy data of CE-SLN were eventually included in the diagnostic accuracy analysis, and five of them also gave the total number of the two groups of SLNs mentioned above, respectively, and the number of metastatic node in each group was included in the metastatic risk ratio analysis. The characteristic data of each study are listed in Table 1, and the quality assessment results of the included studies are shown in Table 2. The final assessment results consider the quality and risk of bias of each study acceptable.

**Table 1:** Characteristics of included studies

Author, year, country	Patients (n)	Age (year) (range and mean)	Invasive cancer/DCIS	Contrast agent	Injection site	Imaging system	Localization method	Comparator
**Maryam, 2015, Iran**	50	23–69	50/0	DEFINITY	Peritumoral parenchyma	Not explained	Guidewire	Blue dye + radio-isotope
**Zhou, 2017, China**	46	35–66	46/0	SonoVue	Periareolar intradermal and peritumoral parenchyma approaching the axilla	MyLab, Twice scanner, A high- frequency linear-array probe (LA522)	Titanium clip	Blue dye
**Li, 2019, China**	453	28–72	453/0	SonoVue	Periareolar intradermal	Philips, iU 22 ultrasound system	Skin marked	Blue dye
**Kenzo, 2017, Japan**	100		94/6	Sonazoid	Periareolar intradermal and subdermal	GE Logiq E9 with XDclear	FNA	Blue dye + radio-isotope
**Tomohiro,2019,Japan**	50		50/0	Sonazoid	Periareolar intradermal and subdermal	Aplio i700®	Guidewire	Blue dye or ICG
**Xie, 2015, China**	101	22–82	101/0	SonoVue	periareolar intradermal	GE Logiq E9 scanners	Guidewire	Blue dye
**Sever, 2011, UK**	80 (2 male)	32–82	78/2	SonoVue	Periareolar intradermal	Acuson Sequoia 512 scanner	Guidewire	Blue dye + radio-isotope
**Kenzo, 2019, Japan**	75	Not explained	66/9	Sonazoid	Periareolar intradermal and subdermal	GE Logiq E9 with XDclear	Skin marked and FNA and 54 US-guided dye marking use ICG	Blue dye + ICG
**Zhao, 2017, China**	110 (1 male)	28–76	95/15	SonoVue	Periareolar intradermal	Acuson S2000	Skin marked	Blue dye
**Zhong, 2018, China**	126 (1 male)	Not explained	110/16	SonoVue	Periareolar intradermal	MyLab Twice system	FNA + skin marked	Blue dye + radio-isotope
**Liu, 2019, China**	75 (1 male)	31–71 (49.3)	66/9	SonoVue	Periareolar intradermal	PhilipsEPIC Q7	Skin marked	Blue dye
**Omoto, 2009, Japan**	20	32–78 (50.9)	16/4	Sonazoid	Subareolar	GE LOGIQ7 BT07	Skin marked	Blue dye, radio-isotope
**Omoto, 2011, Japan, abstract**	181	Not explain	Not explained	Sonazoid	Subareolar	Not explained	Skin marked	Blue dye, radio-isotope
**Cheng, 2014, China, abstract**	80	Not explain	Not explained	SonoVue	Periareolar intradermal	Not explained	Guide wire	Blue dye
**Matsuzawa, 2015, Japan**	32	32–86 (60.4)	26/6	Sonazoid	Subareolar	Aplio500	Skin marked	Blue dye
**Barentsz, 2015, Netherlands**	14 (1 bilateral)	41–68 (56.0)	14/1	SonoVue	Periareolar intradermal and subdermal	Philips iU22 scanner	Placed I-125 seed	Blue dye, radio-isotope

*FNA* fine needle aspiration, *ICG* indocyanine green

**Table 2:**
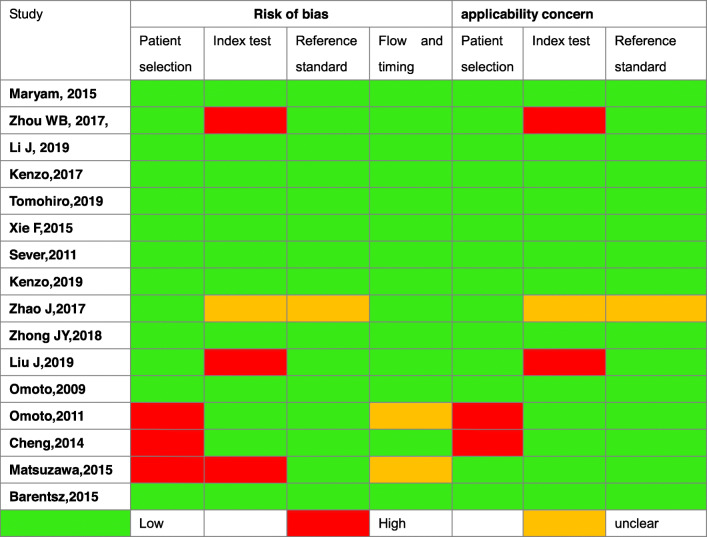
QUADAS-2 risk of bias and applicability concern summary: review authors’ judgements about each domain for each included study

### The localization detection rate of CEUS for SLN

**Fig. 2:**
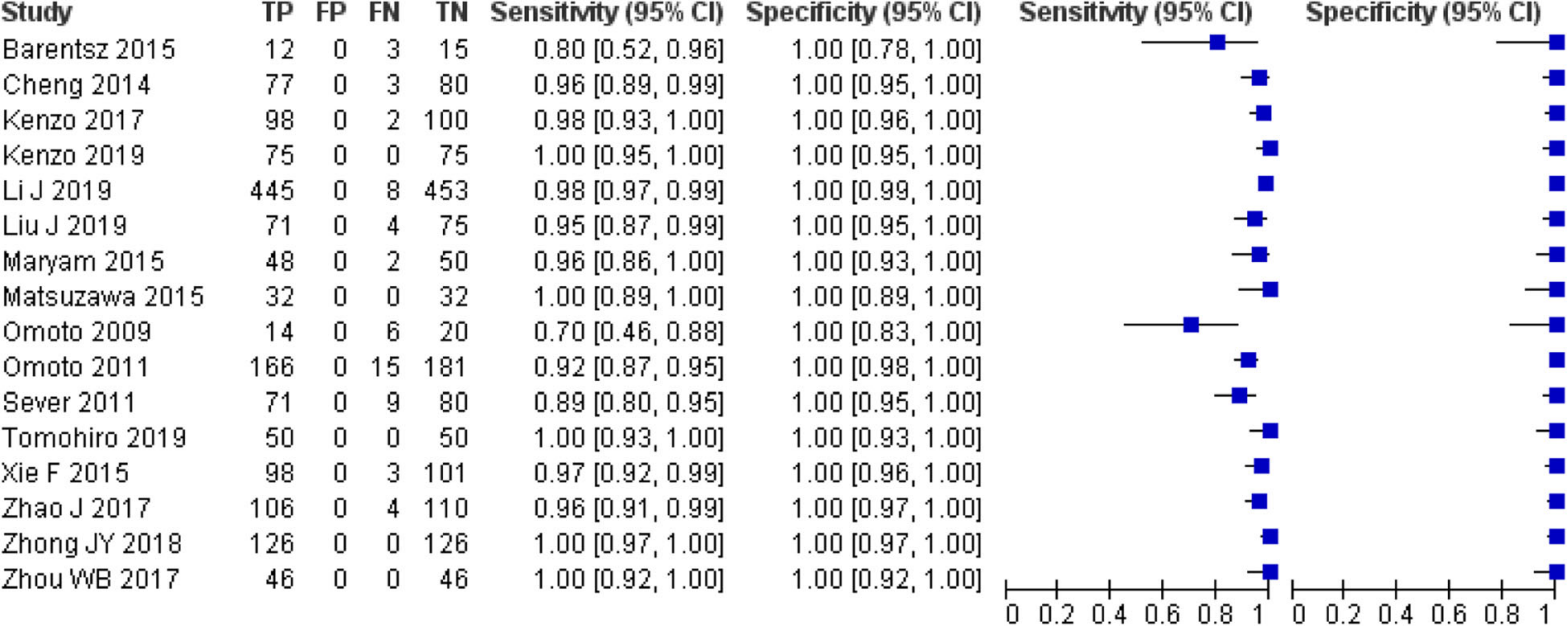
Forest plot of sensitivity and specificity for CEUS-guided SLN detection rate

Of the 16 selected studies, 9 used SonoVue as CEUS material [26–28, 39–44], 6 used Sonazoid [23–25, 45–47], and 1 used DEFINITY [48]. The administration sites and methods of contrast agents mainly include intradermal [23–28, 39–44, 47] and subcutaneous [45, 46] injection around areola or peritumoral parenchyma injection approaching the axilla [48]. SLN localization methods are diversified, including skin labeling [23, 27, 41, 42, 44–47], guidewire localization [25, 26, 40, 43, 48], labeled peptide clip localization [28], iodine-125 seed implantation [39], and ultrasound-guided fluorescent dye injection [24]. Seven studies used the isotope-dye dual tracer method as a comparator [23, 27, 39, 43, 45, 46, 48], while another nine studies were compared with the simple dye method [24–26, 28, 40–42, 44, 47]. Sixteen studies showed that the detection rate of SLN by CEUS method was 70 ~ 100% [23–28, 39–48], as shown in Fig. 2. Due to the differences in the contrast agents, injection methods, criteria, and comparison methods used among the studies, it is suggested that there is great clinical heterogeneity in the methods and indicators of SLN localization detection in various studies. Therefore, it is not appropriate to calculate the pooled SLN detection rate of CEUS.

### Differences in lymph node metastasis rate between CE-SLN and nonCE-SLN

**Fig. 3:**
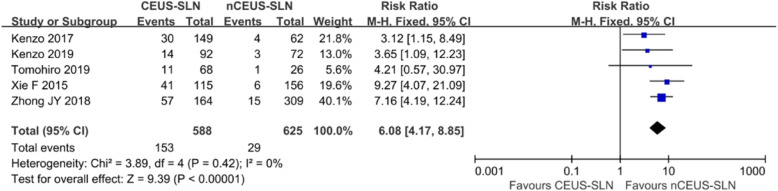
Forest plot of comparison of metastasis risk for CE-SLN and non-CE-SLN

**Fig. 4:**
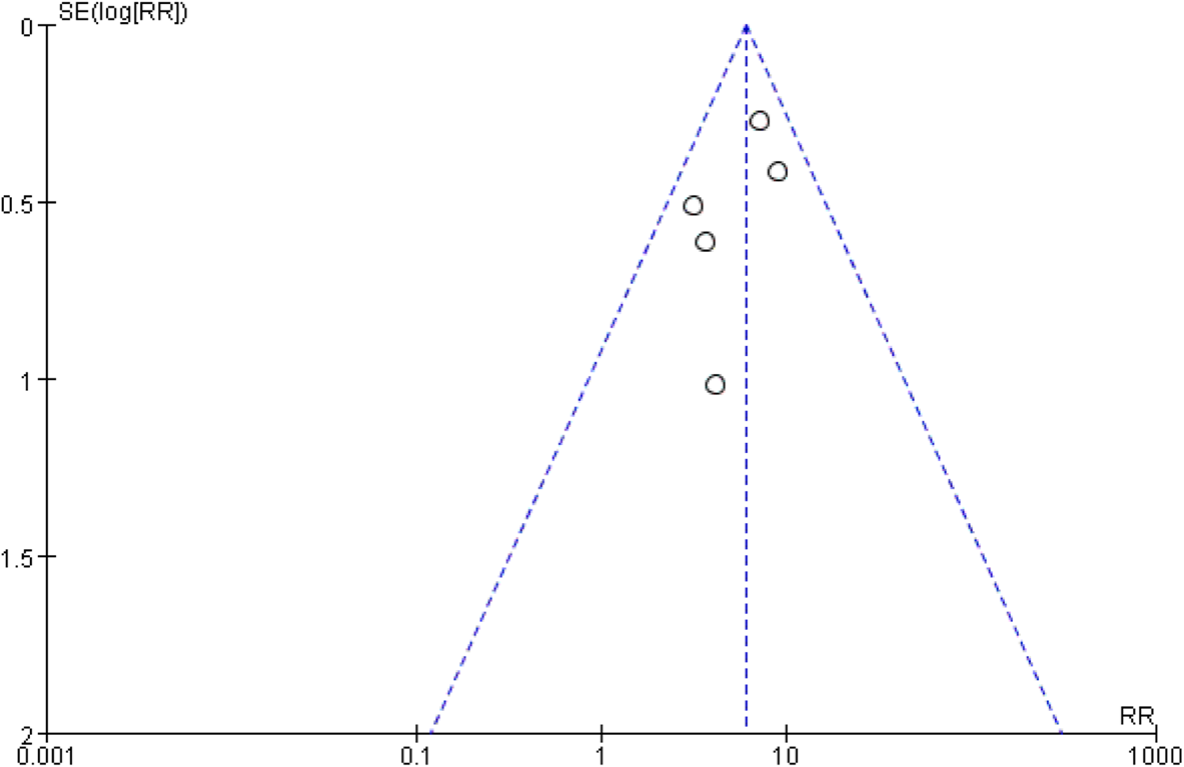
Funnel plot of publication bias between five studies

Five studies [23–27] further reported the total number and metastatic number of CE-SLNs and nonCE-SLNs in successful localization patients, and the pathologic positive criteria adopted in all the studies were the same, including macro- and micro-metastasis, so there is no difference in threshold effect [36]. Three of the studies [23, 24, 27] used skin marked localization and lymph node size, shape, depth, and other parameters as criteria to confirm CE-SLN in surgery, while the other two studies [25, 26] used guidewire localization to confirm CE-SLN. CE-SLN and nonCE-SLN obtained in surgery were sent to pathological examination, respectively. The positive ratio of CE-SLNs was significantly higher than that of nonCE-SLNs, and the data summary is shown in Table 3. Statistical heterogeneity is not observed across the five studies (*I*^2^ = 0.0%). In addition, the assessment of risk of bias in the studies is considered acceptable (Table 2). A fixed-effects model was used to calculate risk ratio (RR) (Review Managers software 5.3), node-positive rate: CE-SLN vs nonCE-SLN, 26.0% vs 4.6%, RR is 6.08 (95% CI 4.17–8.85), as shown in Figs. 3 and 4.

**Table 3:** The differences between CE-SLNs and non-CE-SLNs in pathological state

Author	Year	CE-SLN		nonCE-SLN		Median number of CE-SLN (***n***)	Median number of B/R-SLN (***n***)
		Positive, ***n*** (%)	Negative, ***n*** (%)	Positive, ***n*** (%)	Negative, ***n*** (%)		
**Kenzo**	2017	30 (20.1%)	119 (79.9%)	4 (6.5%)	58 (93.5%)	1	2
**Kenzo**	2019	14 (15.2%)	78 (84.8%)	3 (4.2%)	69 (95.8%)	1	2
**Tomohiro**	2019	11 (16.2%)	57 (83.8%)	1 (3.8%)	25 (96.2%)	1	2
**Xie**	2015	41 (35.7%)	74 (64.3%)	6 (3.8%)	150 (96.2%)	1	2
**Zhong**	2018	57 (34.8%)	107 (65.2%)	15 (4.9%)	294 (95.1%)	1	3

*CE-SLN* sentinel lymph node that identified by contrast-enhanced ultrasound, *nonCE-SLN* sentinel lymph node that not identified by contrast-enhanced ultrasound, *B/R-SLN* sentinel lymph node that identified by blue dye/radionuclide method

### Diagnostic accuracy for CEUS-guided SLNB

**Fig. 5:**
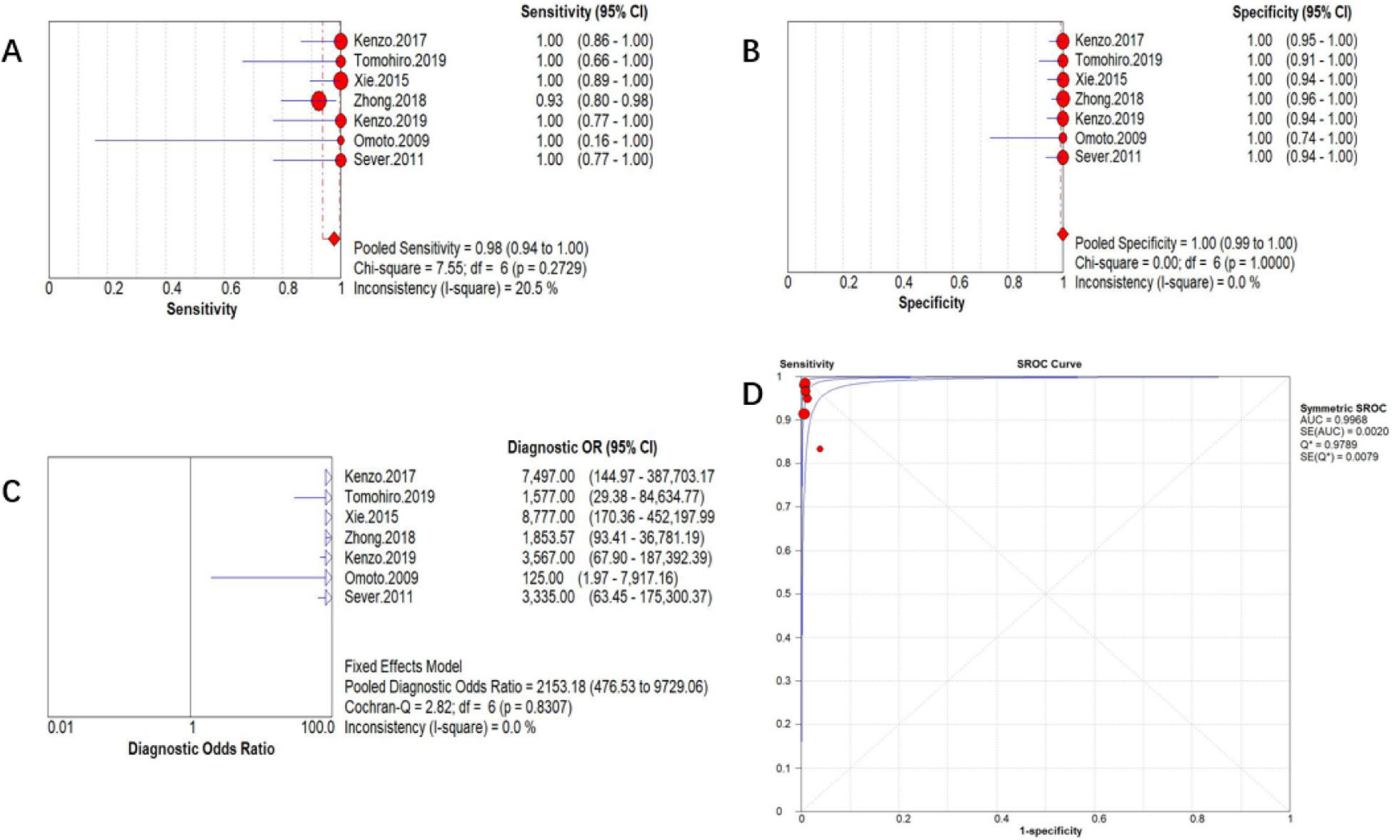
Pooled analysis of the diagnostic accuracy of seven studies. **a** Sensitivity of CE-SLN in diagnosing overall sentinel nodes pathological status. **b** Specificity of CE-SLN in diagnosing overall sentinel nodes pathological status. **c** DOR of CE-SLN in diagnosing overall sentinel nodes pathological status. **d** SROC Curve of CE-SLN in diagnosing overall sentinel nodes pathological status

A total of seven studies [23–27, 43, 45], including the above five studies, provided the detailed pathological status of CE-SLN and nonCE-SLN in individual patient who successfully identified SLN by CEUS. Accordingly, we can summarize the diagnostic accuracy data of these patients, as shown in Table 4. Statistical heterogeneity is mild across the studies(*I*^2^ is from 0.0 to 20.5%). The assessment of risk of bias in the seven studies is considered acceptable (Table 2). So we combined them in a meta-analysis by fixed-effects model (Meta-disc 1.4), and the results show that the combined sensitivity of CE-SLN in diagnosing overall sentinel nodes pathological status is 98% (95% CI 0.94–1.00), specificity is 100% (95% CI 0.99–1.00), diagnostic odds ratio (DOR) is 2153.18 (95% CI 476.53–9729.06), and area under the subject receiver operating characteristic (SROC) curve is 0.9968, as shown in Fig. 5.

**Table 4:** Summarize of the diagnostic accuracy data for seven studies

Author, publication year	CEUS successful patients	Method of axillary preoperative assess	The proportion of patients ≥ T2 or mean T	CE-SLN (+) patients(TP)	CE-SLN (−)/nonCE-SLN (+) patients (FN)	CE-SLN (−)/nonCE-SLN(−) patients (TN)	Accuracy (%)
**Kenzo, 2017**	98	US or MRI no suspicious LN, or FNA proved negative	22%	25	0	73	100
**Tomohiro, 2019**	50	US or MRI no suspicious LN, or FNA proved negative	30%	9	0	41	100
**Xie, 2015**	98	FNA proved LN negative	19.8%	33	0	65	100
**Zhong, 2018**	126	US or MRI no suspicious LN, or FNA proved negative	42.9%	37	3	86	97.6
**Kenzo, 2019**	75	US or MRI no suspicious LN	42.7%	14	0	61	100
**Omoto, 2009**	14	US no suspicious LN	15%	2	0	12	100
**Sever, 2011**	71	US no suspicious LN	Mean *T* = 14 mm	14	0	57	100

*US* ultrasonography, *MRI* magnetic resonance imaging, *LN* lymph node, *TP* true positive, *TN* true negative, *FN* false negative

## Discussion

Previous reports have shown that the detection rate of SLN by CEUS was 70 ~ 100%, but the detection rate less than 90% was reported by a few studies with very small sample size [39, 43, 45], and most of the studies with a sample size greater than 100 [23, 26, 27, 42, 44, 46] had a detection rate of more than 90%. Cox et al. [49] reported a multicentric joint study exploring CEUS-guided core needle biopsy of SLN, which included a total of 1859 patients. The final results showed CEUS detection rate of SLN was 89.2%, and the reason why the detection rate did not reach 90% might be related to the relatively high rate of patients who had received mass resection in the included population. Li et al. [41] recently reported their detection rate of CE-SLN was 98.2% in 453 participating patients, which was similar to the results reported earlier by Sever et al. [50] and Cox et al. [51], suggesting that the detection rate of this method is not inferior to isotope and blue dye.

Although the isotopic method and the blue dye method are considered to be the gold standard of SLNB, the actuality of intraoperative excessively rummage SLN is indeed a considerable problem. Due to the small molecular weight of the blue dye, it is easy to enter the secondary lymphatic vessel or lymph node, which lead to the removal of the non-sentinel lymph node. That may reduce the false negative rate to some extent, but may also bring more postoperative complications. Some studies included in this review summarized the differences between the number of SLNs detected by CEUS and conventional method (isotope and/or blue dye), and the results were consistent. The median of SLN detected by CEUS was 1, while the median of SLN detected by conventional method was 2–3 in the same group of patients. Five studies detailed the number of CE-SLNs and nonCE-SLNs and the number of positive SLNs, respectively. Despite differences in the contrast agents used in these studies, however, the method of operation was almost identical, and the number of enhanced sentinel nodes obtained was very close, with the same median, so it is appropriate to do a meta-analysis of them. The result of the meta-analysis shows that CE-SLNs are six times more positive than nonCE-SLNs (as shown in Fig. 3), suggesting that CE-SLN may be the more accurate sentinel lymph node, so just excising the CE-SLN may be enough.

Tomohiro et al. [25] implemented a study about SLNB guided by CEUS combined with blue dye in patients with early invasive breast cancer. They defined the first lymph node that receives lymphatic drainage from areola detected by both CEUS and blue dye as first-SLN, and other SLNs detected by blue dye alone was defined as downstream-SLNs; the research result showed that when the first-SLN was not detected in cancer metastasis, other downstream-SLNs in the same patient were also negative. In addition, the guidance of guidewire can improve the efficiency of surgical exploration, effectively shorten the surgical incision, narrow the scope of surgery, and shorten the operation time. This meta-analysis also suggests that CE-SLN is highly representative of the pathological status of overall axillary sentinel lymph nodes (as shown in Fig. 5), indicating that the SLNs found by CEUS are more consistent with the concept of sentinel lymph node. Although previous studies implemented by Sever et al. [43] and Omoto et al. [45] showed a low successful rate of detection of CE-SLN, the diagnostic accuracy of CE-SLN in these studies was still inspiring. Zhong et al. [27] reported three patients had negative CE-SLN but positive nonCE-SLN, which was defined as false negative CE-SLN, in their study. This might be due to the certain factors in the process of CEUS, for example, short operating time or excessive subcutaneous fat accumulation, and the neglect of individual CE-SLN. Wang’s study [28] also suggested that the presence of sentinel lymphatics deep within the gland may be overlooked during exploration due to tissue depth and instrumental parameters. Multiple locations injection, repeated operation, and careful CEUS exploration can minimize the occurrence of this condition.

The advantage of CEUS in comparison with blue dye or isotope is that it can intuitively understand the specific location and drainage pattern of sentinel lymph node before surgery and more accurately evaluated the pathological status of SLN through various methods, such as needle biopsy under CEUS localization or contrast enhancement image analysis [41, 42, 44, 49, 50]. Result of a previous meta-analysis [52] showed that CEUS-guided SLN localization and preoperative needle biopsy could eliminate unnecessary SLNB in about 54% of SLN-positive patients. The advantages of this technique may make it more promising for sentinel lymph node biopsy after neoadjuvant chemotherapy. The result of SANTINA study [53] suggested that SLNB in cN0 patients administrated before neoadjuvant chemotherapy lead to loss of the opportunity of SLNB after neoadjuvant therapy for sentinel node-positive patients, which may cause excessive axillary dissection for a considerable number of patients. The detection rate of SLN identified by CEUS is no less than that by blue dye, and the pathological data of SLN can be obtained under the premise of very minimally invasive treatment, which may be the most effective method to solve both localization and pathological evaluation of SLN before neoadjuvant therapy. Surgeon can accurately locate SLN and place marker by CEUS before starting neoadjuvant therapy and perform pathological puncture biopsy on SLN, which may further improve the feasibility of SLNB after neoadjuvant therapy.

The deficiency of these studies is that the sample size is small, and most of all is the very early patients (T0-1). In addition, most patients who had a history of surgery or trauma in the armpit or the outer upper quadrant of the breast were excluded, which inevitably resulted in selective bias, while the accuracy of this method is still uncertain in patients with larger tumors. The study of Wang [28] listed the pathological status of CE-SLN and other lymph nodes in each patient after axillary dissection, and the result showed that the false negative rate of CE-SLN was 4/15 (26.7%). Among the patients included in this report, 62.5% were in stage T2, which led to a higher likelihood of lymph node metastasis [54–56], and the preoperative assessment of axillary status was limited to axillary palpation, while in the absence of imageological or pathological assessment. In 3 of four false negative cases, the existence of discontinuous sentinel lymphatic channels and the non-enhanced SLN was confirmed, which may be the main reason for the increase of final false negative rate, just like several studies [21, 36, 37, 39] exploring patterns of lymph node enhancement, suggesting that such unenhanced sentinel nodes connected with discontinuous lymphatic vessels are most likely to be positive. So SLNB is not recommended in this situation. However, the result of this meta-analysis also suggests that this method may be more suitable for very early-stage patients with small tumors. For patients with early invasive tumor as T1 and below, it is possible to improve the accuracy of targeted SLN localization through CEUS, thereby reducing the need for sentinel lymph node detection and the trauma of axillary surgery. In addition, except for Wang’s report, none of SLN-negative patients received ALND in other studies, so the false negative rate of SLNB guided by CEUS alone or in combination with blue dye cannot be fully determined at present. It is worth looking forward to further exploring the overall axillary lymph node status of these patients in a large sample study.

CEUS-guided preoperative sentinel node localization can be used as an effective tracer. In patients with early-stage breast cancer, especially below T2, combining this method with the conventional method can further improve the accuracy of SLNB. Of course, prospective studies with larger population are needed to confirm this conclusion.

## Data Availability

All data analyzed during this study are included in this published article.

## Abbreviations

SLN: Sentinel lymph node
SLNB: Sentinel lymph node biopsy
CEUS: Contrast-enhanced ultrasound
CE-SLN: SLN identified by CEUS
nonCE-SLN: SLN not identified by CEUS
B/R-SLN: Sentinel lymph nodes identified by blue dye/radionuclide method
ALND: Axillary lymph node dissection
RR: Risk ratio
DOR: Diagnostic odds ratio
SROC: Subject receiver operating characteristic
